# The prognostic evaluation of CA19-9, D-dimer and TNFAIP3/A20 in patients with pancreatic ductal adenocarcinoma

**DOI:** 10.1097/MD.0000000000024651

**Published:** 2021-02-12

**Authors:** Peng Xu, XiaoDong Wang, JianJun Qian, ZhengNan Li, Jie Yao, AMan Xu

**Affiliations:** a Department of General Surgery, The Fourth Affiliated Hospital of Anhui Medical University, Hefei; b Department of Hepatobiliary and Pancreatic Surgery, Northern Jiangsu People's Hospital Affiliated to Yangzhou University, Yangzhou; c Department of General Surgery, The First Affiliated Hospital of Anhui Medical University, Hefei, China.

**Keywords:** CA19-9, D-dimer, pancreatic ductal adenocarcinoma, prognosis, TNFAIP3/A20

## Abstract

This study aimed to explore the significance and prognostic value of serum tumor-associated carbohydrate antigen 19-9 (CA19-9), D-dimer, and tumor necrosis factor alpha-induced protein 3 (TNFAIP3/A20) in patients with pancreatic ductal adenocarcinoma (PDAC).

Our study included 148 patients treated for PDAC at Northern Jiangsu People's Hospital Affiliated to Yangzhou University from January 2012 to December 2016. Cutoff prognostic values were predicted using the receiver operating characteristic (ROC) curve. The Kaplan–Meier method was used to assess the survival rates of patients. Univariate and multivariate COX regression analyses were used to evaluate the prognostic factors.

The recommended cutoff values of neutrophil–lymphocyte rate (NLR), platelet-lymphocyte rate (PLR), CA19-9, and D-dimer were 2.04 (sensitivity, 0.59; specificity, 0.9; area under the ROC curve [AUC], 0.749; *P* < .001), 52.94 (sensitivity, 0.73; specificity, 0.95; AUC, 0.829; *P* < .001), 176.66 U/mL (sensitivity, 0.7; specificity, 0.9; AUC, 0.794; *P* < .001), and 1.18 mg/L (sensitivity, 0.82; specificity, 0.9; AUC, 0.845; *P* < .001), respectively. Positive TNFAIP3/A20 expression was considered as an inclusion criterion. Serum CA19-9 expression was related with lymph node metastasis (*P* = .010), tumor-lymph node-metastasis (TNM) stage (*P* < .001), and survival rate (*P* < .001). D-dimer was correlated with tumor differentiation grade (*P* = .014), tumor size (*P* = .045), TNM stage (*P* < .001), and survival rate (*P* < .001). TNFAIP3/A20 was correlated with tumor differentiation grade (*P* < .001), body mass index (BMI) (*P* < .001), TNM stage (*P* = .014), and survival rate (*P* < .001). Kaplan–Meier curves showed that PDAC patients had significant differences in CA19-9, D-dimer, and TNFAIP3/A20 expressions (*P* < .05). CA19-9, D-dimer, TNM stage, tumor differentiation grade, and TNFAIP3/A20 were independent prognostic markers for PDAC in univariate and multivariate COX analyses.

CA19-9, D-dimer, and TNFAIP3/A20 were found to be independent prognostic markers for PDAC patients.

## 1 Introduction

Pancreatic cancer can be a lethal diagnosis, as it has often metastasized to other organs by the time of detection due to a lack of specific symptoms in its early stages. Although many therapies exist for pancreatic cancer, the 5-year survival rate remains <9%.^[1,2]^ Prognosis is closely associated with early detection and treatment. Currently, pancreatic cancer is diagnosed through imaging or biopsy. However, research into prognostic blood markers such as the neutrophil-lymphocyte rate (NLR) and platelet-lymphocyte rate (PLR) have recently begun.^[3–5]^

Serum carbohydrate antigen 19-9 (CA19-9) and D-dimer are considered prognostic factors for pancreatic cancer.^[6–8]^ CA19-9 is a sialic acid containing glycan antigens found in both glycoproteins and glycolipids,^[7]^ while D-dimer is a soluble fibrin degradation products from the orderly breakdown of thrombi by the fibrinolytic system. D-dimer, fibrinogen, and CA19-9 can be used as indicators of disease recurrence in patients with resectable pancreatic cancer, and preoperative D-dimer can predict survival rate.^[8]^ The study by Watanabe et al found that plasma D-dimer levels can be used as a postoperative prognostic marker for resectable pancreatic cancer.^[9]^ Other studies have found a correlation between D-dimer concentration in portal vein blood and the survival of non-resectable pancreatic cancer patients.^[10]^ D-dimer was also reported as an independent prognostic marker for digestive cancers.^[11,12]^

Tumor necrosis factor alpha-induced protein 3 (TNFAIP3/A20) is a cytoplasmic zinc-finger protein expressed in a variety of cells, and is associated with Crohn's disease, systemic lupus erythematosus, and autoimmune diseases.^[13]^ It interacts with the inflammatory response by regulating the canonical NF-κβ pathway and its termination.^[14]^ Studies have suggested that miR-125a, a mircoRNA involved in post-transcription regulation of gene expressions, promotes chemo-resistance to gemcitabine in pancreatic cells by targeting TNFAIP3/A20, found to be reduced in pancreatic cancer tissues, strongly correlating with pancreatic cancer behavior.^[15,16]^

Although CA19-9 is a marker of pancreatic tumors, external factors such as jaundice and other inflammatory factors can influence its accuracy.^[17,18]^ D-dimer and TNFAIP3/A20 have also been studied in cancer.^[11,16]^ As a stress response gene in endothelial cells (ECs), TNFAIP3/A20 has a protective effect against tumor necrosis factor (TNF)-mediated apoptosis, and inhibits inflammation.^[19,20]^ D-dimer level is associated with vascular EC injury.^[21]^ A relationship between TNFAIP3/A20, D-dimer and CA19-9 may hence exist. The prognostic value of CA19-9, D-dimer, and TNFAIP3/A20 in patients with pancreatic ductal adenocarcinoma (PDAC) have not been thoroughly and extensively discussed. Our study therefore aimed to explore the relationships of these three indicators with the characteristics and survival of patients with PDAC.

## 2 Materials and methods

### 2.1 Inclusion and exclusion criteria

This study was approved by the Ethics Committee of Northern Jiangsu People's Hospital Affiliated to Yangzhou University. Written informed consent was obtained from all patients. This study was performed according to the Statement of Helsinki.

Among the inclusion criteria were

1. patients pathologically diagnosed with PDAC;
2. those who did not receive radiotherapy and/or adjuvant chemotherapy before surgical resection;
3. patients with definite indications for surgical treatment; and
4. those with peripheral blood tests performed within 1 week of operation.

Exclusion criteria included

1. patients previously diagnosed with other primary tumors;
2. patients concurrently diagnosed with diseases affecting peripheral blood cell counts, including infections;
3. patients with tumors previously treated with radiotherapy and/or adjuvant chemotherapy; and
4. those who died within 4 weeks of the procedure.

### 2.2 Patient characteristics and tissue specimens

Our study included 148 patients with PDAC who were subjected to surgical resection the hospital from January 2012 to December 2016. All tumors investigated were resectable, and baseline patient data including blood test results and pathological features were collected (Table 1). Disease progression was classified according to the American Joint Committee on Cancer Staging System. Peripheral blood tests were performed within 1 week of the procedure. Prognostic value of NLR and PLR in PDCA were analyzed. The recommended cutoff values of CA19-9, D-dimer, PLR, and NLR before the resection were determined by the Youden's index of the receiver operating curve (ROC) (maximum [sensitivity + specificity – 1]).

**Table 1:** Patient characteristics.

Characteristic	Median (25th–75th percentile) or No. (%)
Gender	
Male	85 (57.4)
Female	63 (42.6)
Age (year)	
<60	47 (31.8)
≥60	101 (68.2)
Smoking	
Yes	72 (48.6)
No	76 (51.4)
Alcohol consumption	
Yes	57 (38.5)
No	91 (61.5)
Diabetes	
Yes	56 (37.8)
No	92 (62.2)
High blood pressure	
Yes	26 (17.6)
No	122 (82.4)
BMI	
18.5 ≤ BMI < 24	105 (70.9)
BMI < 18.5, 24 ≤ BMI	43 (29.1)
Lymph node metastasis	
N1	104 (70.3)
N0	44 (29.7)
TNM stage	
I	31 (20.9)
II+III	117 (79.1)
Tumor size	
>5cm	39 (26.4)
≤5cm	109 (73.6)
Tumor differentiation grade	
Low	50 (33.8)
Moderate	78 (52.7)
High	20 (13.5)
Tumor volume (cm^3^)	36.65 (13.34–66.64)
Neutrophil count	5.30 (4.6–5.8) × 10^9^/L
Platelet count	182.5 (104.25–255.75) × 10^9^/L
Lymphocyte count	2.5 (2.10–2.90) × 10^9^/L
TNFAIP3/A20	
Positive	37 (25.0)
Negative	111 (75.0)
CA19-9 U/mL	271.54 (81.08–453.34)
D-dimer mg/L	3.17 (0.98–5.41)

### 2.3 Immunohistochemical analysis

According to standard procedures of immunohistochemistry, tissues of all 148 patients with pathologically diagnosed PDCA were fixed in formalin, and were subsequently embedded in paraffin, cut into 4-μm thick sections, dewaxed, and hydrated. Sections were then rinsed with 10 μmol/L citrate buffer, microwaved for 10 min. Sections were then immersed in 3% hydrogen peroxide and anhydrous methanol, and were treated with 10% goat serum albumin for 20 min. A 1:100 diluted primary antibody for TNFAIP3/A20 protein (rabbit polyclonal antibody, Abcam, USA) was added, and samples were stored at 4°C overnight before being added to a secondary antibody at room temperature. The sections were incubated with horseradish peroxidase-conjugated streptavidin for 1 h, and were then developed with diaminobenzidine (DAB). Finally, the sections were counterstained with Mayer's hematoxylin.

To evaluate the expression of TNFAIP3/A20, each section was randomly graded based on the average percentage of positive cells (P) and the intensity of staining (S) in five areas (per100 fields of view). The percentage score of P was categorized as 0 (<5%), 1 (5–24%), 2 (25–49%), 3 (<50–75%), and 4 (> 75%); while S was classified as 0 (no staining), +1 (weak), +2 (medium), and +3 (strong). The final score was obtained by the following algorithm: SCORE = Σ (P × S). The grading standards were as follows: 0 (−), 1 – 3 (+), 4 – 7 (++), and 8 – 12 (+++).

### 2.4 Follow-up and treatment

Data of 162 PDCA patients were initially collected. Following resection, patients with poor health were given supportive therapy, while the remaining received chemotherapy or other targeted treatment. As first-line chemotherapy, 96 patients received gemcitabine plus capecitabine, while 66 received oral S-1. Both regimes were repeated every 3 weeks for 6 cycles. Twelve patients who were found with tumor recurrence or tumor progression were switched to the gemcitabine plus erlotinib. No patients received adjuvant radiotherapy. Follow-up data were obtained every month by telephone and outpatient services. The follow-up period ended in December 2018, during which 8 and 6 patients were excluded due to lost contact and death from non-cancer diseases, respectively. Overall, 148 patients were included.

### 2.5 Statistical explanation

Statistical analysis was performed using the SPSS 23.0 software (SPSS Inc, Chicago, IL). Cutoff values of indexes were determined according to the Youden's index of the ROC curve (maximum [sensitivity + specificity – 1]). Area under the ROC curve (AUC) was compared using the *z*-test. Categorical variables were analyzed using either the Pearson χ^2^ test or the Fisher's exact test. For survival analyses, either the Kaplan–Meier method with log-rank test or univariate and multivariate Cox regression methods were used. *P* < 0.05 was defined as statistically significant.

## 3 Results

### 3.1 Patient characteristics

Among the included patients, 85 (57.4%) were male and 63 (42.6%) were female. Median age was 63 years (range, 34–81 years). Median follow-up period was 12 months (range, 2–28 months). Tumors of >5 cm were found in 39 (26.4%) patients; pathological stage I was observed in 31 (20.9%) patients, while stages II–III were observed in 117 (79.1%) patients. Low, moderate, and high tumor differentiation levels were observed in 50, 78, and 20 cases, respectively. (Table 1)

### 3.2 Cutoff prognostic values predicted by the ROC curve

**Figure 1:**
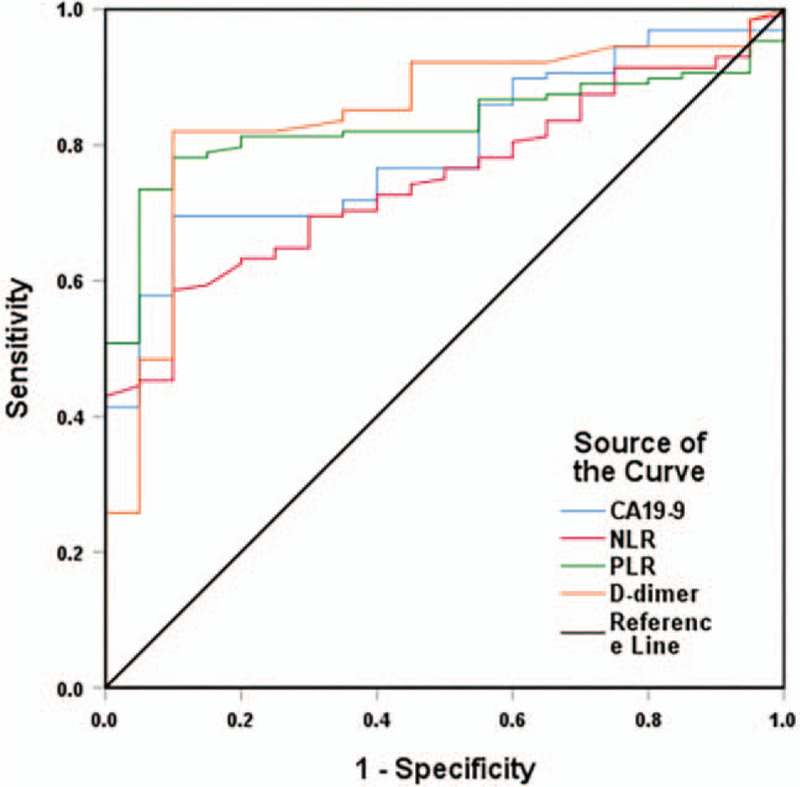
ROC curves of prognostic markers in PDAC patients.

Based on the Youden's index, the recommended cutoff values of NLR, PLR, CA19-9, and D-dimer were 2.04 (sensitivity, 0.59; specificity, 0.9; AUC, 0.749; *P* < .001), 52.94 (sensitivity, 0.73; specificity, 0.95; AUC, 0.829; *P* < .001), 176.66 U/mL (sensitivity, 0.7; specificity, 0.9; AUC, 0.794; *P* < .001), and 1.18 mg/L (sensitivity, 0.82; specificity, 0.9; AUC, 0.845; *P* < .001), respectively (Fig. 1).

### 3.3 Relationship between the cutoff values of CA19-9, D-dimer, and TNFAIP3/A20 with clinicopathologic characteristics

**Figure 2:**
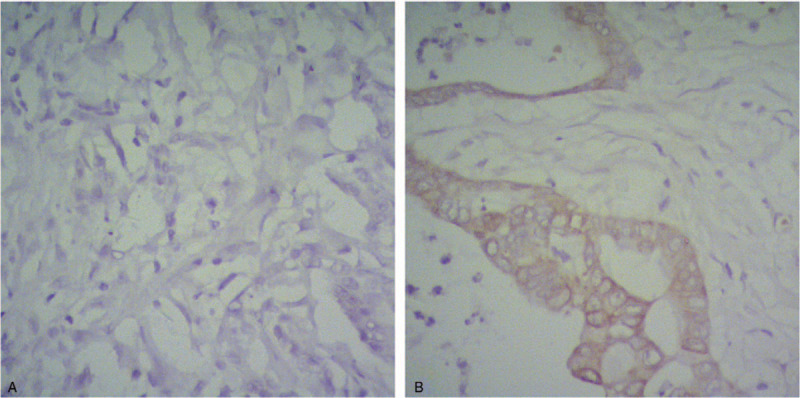
Expression of TNFAIP/A20 in PDAC. (A) Negative TNFAIP/A20 expression in PDAC (X400). (B) Positive TNFAIP/A20 expression in PDAC (×400).

The cutoff value of CA19-9, as determined by the Youden's index, showed no significant association with age, gender, tumor size, tumor differentiation grade, smoking, alcohol consumption, diabetes, high blood pressure, or BMI (*P* > .05). However, statistically significant associations were observed between CA19-9 cutoff value and lymph node metastasis (*P* = .010), TNM stage (*P* < .001), and survival rate (*P* < .001). D-dimer cutoff value was statistically associated with tumor differentiation grade (*P* = .014), tumor size (*P* = .045), TNM stage (*P* < .001), and survival rate (*P* < .001), with no statistical differences shown with gender, age, smoking, alcohol consumption, diabetes, high blood pressure, BMI, or lymph node metastasis (*P* > .05). Reduced TNFAIP3/A20 expression was seen in 25.0% (37/148) of PDAC tissues (Fig. 2), which significantly associated with tumor differentiation grade (*P* < .001), BMI (*P* < .001), TNM stage (*P* = .014), and survival rate (*P* < .001). No relationship was observed between TNFAIP3/A20 and gender, age, smoking, alcohol consumption, diabetes, high blood pressure, or lymph node metastasis (*P* > .05) (Table 2).

**Table 2:** Relationship between cutoffs of CA19-9, D-dimer and TNFAIP3/A20 with clinicopathologic characteristics.

	CA19-9 (U/mL)			D-dimer (mg/L)			TNFAIP3/A20		
Patient-related factors	<176.66 (n = 57)	≥176.66 (n = 91)	*P*	<1.18 (n = 41)	≥1.18 (n = 107)	*P*	Positive (n = 37)	Negative (n = 111)	*P*
Gender			.265			.589			.502
Male	36	49		25	60		23	62	
Female	21	42		16	47		14	49	
Age (years)			.137			.425			.126
<60	14	33		11	36		8	39	
≥60	43	58		30	71		29	72	
Tumor sizes			.439			.045			.914
<5 cm	44	65		35	74		27	82	
≥5 cm	13	26		6	33		10	29	
Tumor differentiation grade			.141			.014			<.001
Low	14	36		8	42		1	49	
Moderate	33	45		23	55		25	53	
High	10	10		10	10		11	9	
Survival rate			<.001			<.001			<.001
Yes	18	2		18	2		13	7	
No	39	89		23	105		24	104	
Smoking			.805			.279			.255
Yes	27	45		17	55		21	51	
No	30	46		24	52		16	60	
Alcohol consumption			.477			.226			.380
Yes	24	33		19	38		12	45	
No	33	58		22	69		25	66	
Diabetes			.585			.341			.117
Yes	20	36		13	43		18	38	
No	37	55		28	64		19	73	
High blood pressure			.077			.386			.803
Yes	14	12		9	17		6	20	
No	43	79		32	90		31	91	
BMI			.364			.439			<.001
18.5 ≤ BMI < 24	38	67		31	74		35	70	
BMI < 18.5, BMI ≥ 24	19	24		10	33		2	41	
Lymph node metastasis			.010			.939			.213
N1	47	57		29	75		29	75	
N0	10	34		12	32		8	36	
TNM stage			<.001			<.001			.014
I	21	10		19	12		13	18	
II+III	36	81		22	95		24	93	

### 3.4 Relationship of CA19-9, D-dimer, and TNFAIP3/A20 with prognosis

**Figure 3:**
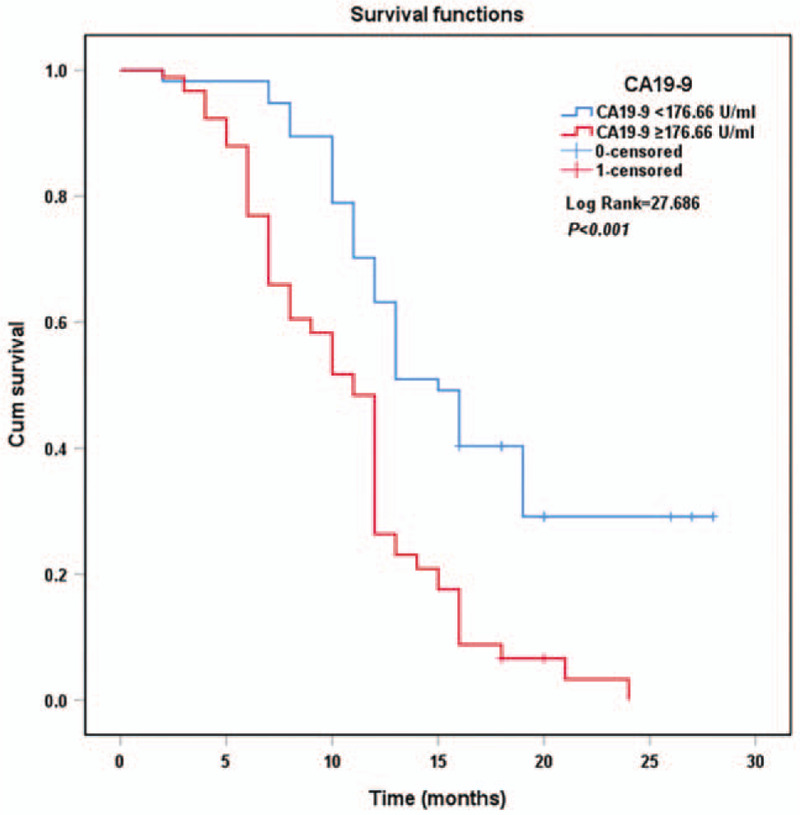
Kaplan–Meier curves of survival in patients according to CA19-9 expression.

**Figure 4:**
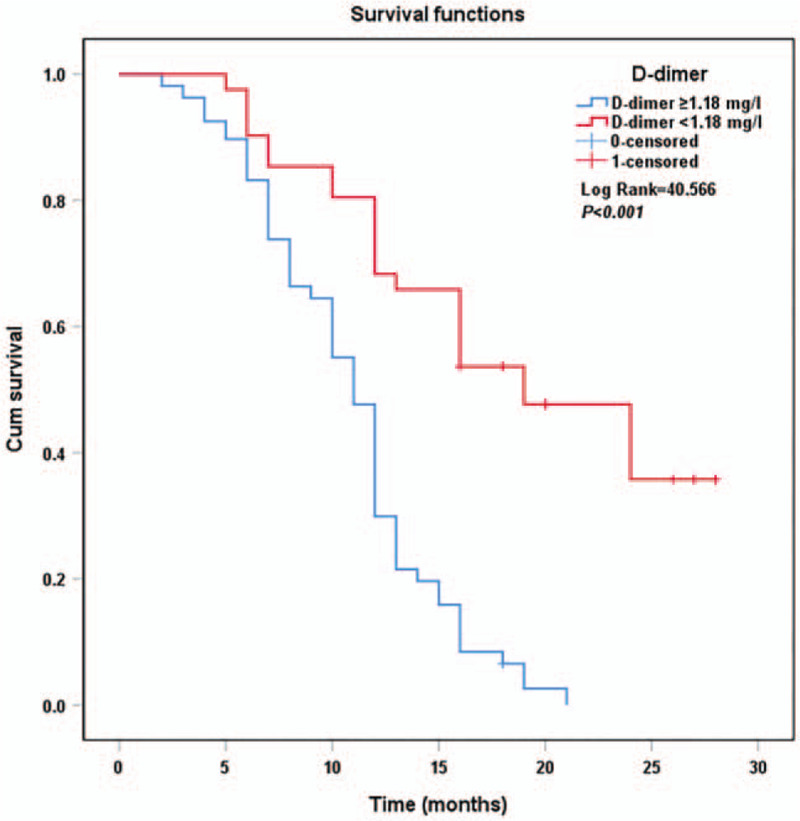
Kaplan–Meier curves of survival in patients according to D-dimer expression.

**Figure 5:**
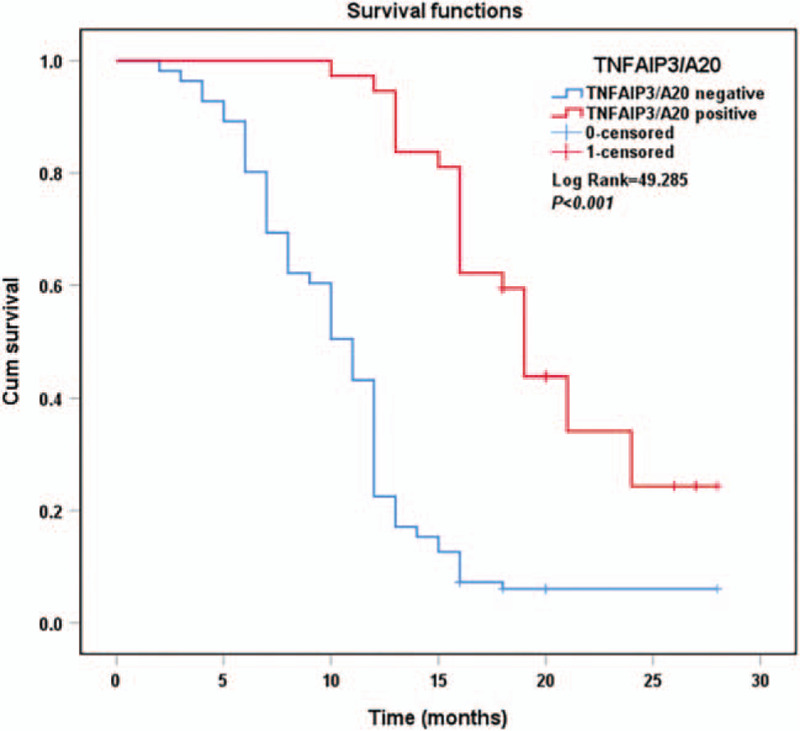
Kaplan–Meier curves of survival in patients according to TNFAIP/A20 expression.

The Kaplan–Meier survival curves for PDAC patients stratified by CA19-9 (Fig. 3), D-dimer (Fig. 4), and TNFAIP3/A20 expressions (Fig. 5) are shown. The overall survival of patients with CA19-9 < 176.66 U/mL (*P* < .001), D-dimer < 1.18 mg/L (*P* < .001), and positive TNFAIP3/A20 expression (*P* < .001) was longer than that of their counterparts. Multivariate Cox analysis identified CA19-9 (*P* = .005), D-dimer (*P* = .005), TNFAIP3/A20 (*P* < .001), TNM stage (*P* = .007), and tumor differentiation grade (*P* < .001) as independent risk factors for overall survival (Table 3).

**Table 3:** Proportional risk model for predicting overall survival.

	Univariate COX analysis		Multivariate COX analysis	
Characteristic	HR value (95% CI)	*P*	HR value (95% CI)	*P*
Gender	1.208 (0.852-1.714)	.289		
Age (years)	0.821 (0.565–1.193)	.302		
Tumor sizes	1.306 (0.890–1.916)	.172		
Tumor differentiation grade	0.403 (0.3–0.541)	<.001	0.573 (0.419–0.783)	<.001
Lymph node metastasis	1.535 (1.053–2.237)	.026	1.266 (0.851–1.884)	.244
Tumor volume	1.0039 (0.989–1.018)	.646		
Smoking	0.957 (0.674–1.359)	.804		
Alcohol consumption	0.912 (0.635–1.310)	.617		
Diabetes	1.084 (0.761–1.544)	.656		
High blood pressure	1.379 (0.869–2.188)	.172		
BMI	1.30 (0.883–1.914)	.184		
TNM stage	4.454 (2.699–7.352)	<.001	2.228 (1.240–4.003)	.007
NLR	1.420 (1.107–1.820)	.006	1.024 (0.722–1.453)	.892
PLR	1.007 (1.004–1.010)	<.001	1.004 (0.999–1.009)	.131
TNFAIP3/A20	0.227 (0.141–0.365)	<.001	0.357 (0.209–0.604)	<.001
CA199 ≥ 176.66 U/mL	2.530 (1.722–3.716)	<.001	1.827 (1.205–2.770)	.005
D-dimer ≥ 1.18 mg/L	3.964 (2.436–6.449)	<.001	2.120 (1.252–3.588)	.005

## 4 Discussion

PDAC is a highly malignant form of cancer. Several biomarkers have been proposed for use as identifiers of early disease, as most cases are currently discovered only after widespread metastasis. Treatment involves surgical resection; however, tumor cell spread is often terminal by the time such a treatment option is offered. Identifying biomarkers for early diagnosis of pancreatic cancer is therefore an important topic for future research.^[22–24]^

Although progress has been made in immunotherapy and targeted therapy of pancreatic cancer,^[25,26]^ surgery is still the most common therapy for PDAC. However, due to the limitations of diagnostic techniques, early detection of pancreatic cancer is usually difficult, leading to poor prognosis.^[23]^ It has been shown that some markers may be related to the prognosis, tumor differentiation grade, and TNM stage of PDAC patients.^[6,27]^ Given that these prognostic factors are difficult to be determined preoperatively, studies on preoperative serum markers have become a research hot spot.^[11,28]^

As an independent prognostic indicator, CA19-9 has been attracting increasing attention.^[28]^ CA19-9 levels may be affected by the presence of jaundice and inflammation secondary to PDAC.^[17,18]^ In our study, patients with preoperative CA19-9 ≥ 176.66 U/mL positively correlated with lymph node metastasis (*P* = .010), TNM stage (*P* < .001), and survival rate (*P* < .001), showing a high degree of malignancy. Engle et al. found that CA19-9 expression resulted in hyperactivation of epidermal growth factor receptor (EGFR) signaling, which interacts with the KrasG12D oncogene to produce aggressive pancreatic cancer cells, indicating that CA19-9 may be a useful therapeutic target.^[29]^ We can infer that CA19-9 is related to the malignant degree of pancreatic cancer, possibly through EGFR signaling and its synergistic effects with KrasG12D. However, the mechanism of CA19-9 and tumor progression still remains unclear, and further studies are warranted.

D-dimer is a degradation product of the plasmin-mediated breakdown of cross-linked fibrin polymers. In our study, patients with preoperative D-dimer ≥ 1.18 mg/L was correlated with tumor differentiation grade (*P* = .014), tumor size (*P* = .045), TNM stage (*P* < .001), and survival rate (*P* < .001). The fibrinolytic system promotes tumor growth through angiogenesis, and through the inhibition of apoptosis, tumor cell proliferation and extracellular matrix degradation.^[30]^ Apart from being a marker for activated coagulation and fibrinolysis, D-dimer also plays an important role in cancer progression, invasion, and prognosis.^[12,31]^ Tuan Anh et al found that D-dimer expression positively correlated with that of EFGR.^[32]^ In addition, the EGFR signaling pathway has been shown to regulate apoptosis and the life cycle of pancreatic cancer cells.^[33]^ According to these two studies, we can conclude that D-dimer is related to the malignant degree of tumors through the EGFR signaling pathway. Recent research has shown that activation of the coagulation system is associated with tumorigenesis, as well as tumor cell dissemination and transfer.^[34,35]^ Fibrinolytic enzymes play an important role in tumor invasion and penetration into the circulation. Studies have also shown that tumors are encapsulated in a network structure, and with disease progression and acceleration of metastasis, the network structure gets destroyed and the release of D-dimer increases.^[34,35]^ This also contributes to the increased predisposition to thrombotic diseases in cancer. Changes of D-dimer level may hence be related to the development and progression of tumors.

TNFAIP3/A20 was found to correlate with tumor differentiation grade (*P* < .001), BMI (*P* < .001), TNM stage (*P* = .014), and survival rate (*P* < .001). Kang et al demonstrated that TNFAIP3/A20 plays a role in maintaining mitochondrial function.^[36]^ In contrast, Akbari et al demonstrated that intact mitochondrial function positively correlated with tumor differentiation.^[37]^ From these two studies, we may conclude that TNFAIP3/A20 correlates with the differentiation grade of tumors through maintaining mitochondrial function. As a cytoplasmic zinc-finger protein, TNFAIP3/A20 is reported to be expressed in a variety of human cells, such as T and B lymphocytes, and regulate the dynamic immune response by negatively mediating transcription factor NF-κβ activities and proinflammatory gene expressions.^[13,38–41]^ ENOL-specific TH17 cells have been found to have specific anti-cancer effector functions in pancreatic cancer patients.^[25]^

TNFAIP3/A20 participates in not only the endothelial cell stress response, but also the inflammatory response,^[14,20,42]^ which may affect the expression of CA19-9.^[29]^ Woei-A-Jin et al proved that the binding of CA19-9 to apomucins correlated with microparticle-associated tissue factor (TF) activity.^[43]^ TF expressed by tumor cells triggers the formation of thrombin, which leads to both coagulation and platelet activation,^[44]^ which may then result in an increase in D-dimers. These studies partly explain the relationship between CA19-9, D-dimer, and TNFAIP3/A20; however, their mechanisms still remain unclear, and represent a topic for future research.

The overall survival of patients with CA19-9 < 176.66 U/mL (*P* < .001), D-dimer < 1.18 mg/L (*P* < .001), and positive TNFAIP3/A20 expression (*P* < .001) was longer according to the Kaplan–Meier curves. Previous studies have shown that plasma CA19-9 and D-dimer levels correlated with poor prognosis.^[7,11,12]^ TNFAIP3/A20 was found to be a prognostic factor for human cholangiocarcinoma.^[45]^ Multivariate COX analyses showed that TNM stage (*P* = .007), tumor differentiation grade (*P* < .001), CA19-9 (*P* = .005), D-dimer (*P* = .005), and TNFAIP3/A20 (*P* < .001) were independent prognostic markers in patients with PDAC, suggesting their usefulness in evaluating the prognosis and progression of PDAC.

TNM stage and tumor differentiation grade are not easily evaluated by imaging before surgery; however, preoperative CA19-9 and D-dimer levels are simple to obtain. In clinical practice, sufficient preoperative evaluation and preparation of PDAC patients need to be done to achieve optimal individualized treatment plans. For example, evaluating serum CA19-9 and D-dimer expression levels before the resection may help determine the malignant degree of the tumor and the prognosis of the patient. Detection of TNFAIP3/A20 expression after surgical interventions suggests activated tumor inflammatory reactions and immune microenvironment changes which relate to prognosis and malignancy of patients. For patients with high CA19-9 and D-dimer levels before surgery, early interventions should be considered. In addition to using CA19-9 and D-dimer as preoperative prognostic markers, TNFAIP3/A20, TNM stage, and tumor differentiation grade should also be considered comprehensively after the surgery. Follow-up should be done early to detect recurrence or metastasis, and to prolong the survival of patients. By evaluating relevant indexes before and after surgery, optimal comprehensive treatment strategies for PDAC patients may be formulated, and improve the prognosis of patients. Such proposal, however, requires further research.

We found that CA19-9, D-dimer, and TNFAIP3/A20 carry a prognostic value in PDAC. Patients with a combination of high preoperative serum CA19-9 and D-dimer, with negative TNFAIP3/A20 expression associated with high rates of malignancy and poor prognosis. However, this was a single-center observational study involving a small sample size, and postoperative survival time may have been affected by the choice of postoperative chemotherapy. Large-sample clinical randomized controlled studies are hence needed for further evaluation.

In conclusion, preoperative serum CA19-9 and D-dimer levels and postoperative TNFAIP3/A20 expression reflect the TNM staging of pancreatic cancer. The overall prognosis of patients with high preoperative serum CA19-9 and D-dimer and negative TNFAIP3/A20 expression was poor. CA19-9, D-dimer, and TNFAIP3/A20 are hence considered independent prognostic markers for PDAC patients.

## Author contributions

Peng Xu, JieYao, and AMan Xu carried out the main work, and contributed equally to the study design, as well as drafting and revision of the manuscript. XiaoDong Wang, JianJun Qian, and ZhengNan Li performed the analysis. All authors read and approved of the final manuscript.

**Conceptualization:** Peng Xu, JianJun Qian, Jie Yao, Aman Xu.

**Data curation:** Peng Xu, XiaoDong Wang, Jie Yao, Aman Xu.

**Formal analysis:** Peng Xu, XiaoDong Wang, Aman Xu.

**Funding acquisition:** Peng Xu, JianJun Qian, Jie Yao, Aman Xu.

**Investigation:** Peng Xu, ZhengNan Li, Jie Yao, Aman Xu.

**Methodology:** Peng Xu, XiaoDong Wang, JianJun Qian, ZhengNan Li, Aman Xu.

**Project administration:** Peng Xu, ZhengNan Li, Jie Yao, Aman Xu.

**Resources:** Peng Xu, XiaoDong Wang, JianJun Qian, ZhengNan Li, Jie Yao, Aman Xu.

**Software:** Peng Xu, ZhengNan Li, Jie Yao, Aman Xu.

**Supervision:** Peng Xu, Jie Yao, Aman Xu.

**Validation:** Peng Xu, Jie Yao, Aman Xu.

**Visualization:** Peng Xu, Aman Xu.

**Writing – original draft:** Peng Xu, Jie Yao, Aman Xu.

**Writing – review & editing:** Peng Xu, JianJun Qian, Jie Yao, Aman Xu.

## Abbreviations

Abbreviations: AUC = area under the ROC curve, BMI = body mass index, CA19-9 = serum tumor-associated carbohydrate antigen 19-9, CI = confidence interval, DAB = diaminobenzidine, ECs = endothelial cells, EGFR = epidermal growth factor receptor, HR = hazard ratio, NLR = neutrophil-lymphocyte rate, PDAC = pancreatic ductal adenocarcinoma, PLR = platelet-lymphocyte rate, ROC curve = receiver operating characteristic curve, TF = tissue factor, TNFAIP3/A20 = tumor necrosis factor alpha-induced protein 3, TNM = tumor lymph node metastasis.
